# Future Orientation, Social Support, and Psychological Adjustment among Left-behind Children in Rural China: A Longitudinal Study

**DOI:** 10.3389/fpsyg.2017.01309

**Published:** 2017-08-02

**Authors:** Shaobing Su, Xiaoming Li, Danhua Lin, Maoling Zhu

**Affiliations:** ^1^Institute of Developmental Psychology, Faculty of Psychology, Beijing Normal University Beijing, China; ^2^Institute for Applied Research in Youth Development, Eliot-Pearson Department of Child Study and Human Development, Tufts University Medford, MA, United States; ^3^Arnold School of Public Health, University of South Carolina Columbia, SC, United States; ^4^Center for Foreign Literature Culture, University of Foreign Studies Guangzhou, China

**Keywords:** left-behind children, future orientation, social support, psychological adjustment, longitudinal study

## Abstract

Existing research has found that parental migration may negatively impact the psychological adjustment of left-behind children. However, limited longitudinal research has examined if and how future orientation (individual protective factor) and social support (contextual protective factor) are associated with the indicators of psychological adjustment (i.e., life satisfaction, school satisfaction, happiness, and loneliness) of left-behind children. In the current longitudinal study, we examined the differences in psychological adjustment between left-behind children and non-left behind children (comparison children) in rural areas, and explored the protective roles of future orientation and social support on the immediate (cross-sectional effects) and subsequent (lagged effects) status of psychological adjustment for both groups of children, respectively. The sample included 897 rural children (*M*_*age*_ = 14.09, *SD* = 1.40) who participated in two waves of surveys across six months. Among the participants, 227 were left-behind children with two parents migrating, 176 were with one parent migrating, and 485 were comparison children. Results showed that, (1) left-behind children reported lower levels of life satisfaction, school satisfaction, and happiness, as well as a higher level of loneliness in both waves; (2) After controlling for several demographics and characteristics of parental migration among left-behind children, future orientation significantly predicted life satisfaction, school satisfaction, and happiness in both cross-sectional and longitudinal regression models, as well as loneliness in the longitudinal regression analysis. Social support predicted immediate life satisfaction, school satisfaction, and happiness, as well as subsequent school satisfaction. Similar to left-behind children, comparison children who reported higher scores in future orientation, especially future expectation, were likely to have higher scores in most indicators of psychological adjustment measured at the same time and subsequently. However, social support seemed not exhibit as important in the immediate status of psychological adjustment of comparison children as that of left-behind children. Findings, implications, and limitations of the present study were discussed.

## Introduction

The rapid urbanization in China has led to an unprecedented growth of economically driven rural-to-urban migration, with millions of rural residents migrating and seeking employment in cities (Wen et al., 2015). However, most migrants are not able to receive an urban “Hukou,” an official household residency permit that grants access to social services, including education, healthcare, and the right to buy homes. As a result of the restriction on household registration and education opportunities as well as high living expenses in cities, a huge number of rural children are not able to migrate with their parent(s) and left behind in the rural areas with their grandparents or relatives etc. (Wen et al., 2015). A recent national survey showed that by the end of 2010, over 61 million children in China were left behind due to parental migration, accounting for 21.88% of the child population in China (China Women's Federation, 2013). This figure was almost three times of the number (20 million) estimated in 2000 (China Women's Federation, 2008; Stocktaking Report, 2011), and has increased about 2.42 million from 2008 to 2013 (China Women's Federation, 2013).

Existing studies have indicated that parental migration had negative impacts on children's psychological adjustment (Wen and Lin, 2012). Prior studies showed that left-behind children were likely to suffer depression, anxiety, and loneliness (Magwaza, 1994; Fan and Sang, 2005; Ren and Shen, 2008; Fan et al., 2009), and report low levels of life satisfaction and happiness (Fan et al., 2009; Fan and Zhao, 2010; Gao, 2010; Liu and Ouyang, 2010). However, mixed findings exist regarding the differences in psychological adjustment between left-behind children and comparison children (non-left-behind children in rural areas). For instance, some studies showed that comparison children had better psychological outcomes than left-behind children (Gao et al., 2010; Su et al., 2013), whereas other studies found no differences in certain mental health outcomes (e.g., school satisfaction and happiness) between left-behind and comparison children (Zhang et al., 2006; Hu et al., 2008; Yi and Wu, 2010).

In addition, most existing research considered left-behind children as a single group, ignoring the differences in patterns of parental migration, duration of parental migration, and characteristics of caregivers among different migrant families. A few studies that have differentiated children with one parent migrating from children with both parents migrating did not control for some important characteristics of parental migration and produced mixed results (Zhou et al., 2005; Fan et al., 2009; Sun et al., 2010). For example, Sun et al. (2010) found that compared to children with one parent migrating or children with no parent migrating, children with two parents migrating experienced more loneliness. However, other studies indicated that loneliness did not differ by patterns of parental migration (e.g., Zhou et al., 2005). To better understand how parental migration affects children's psychological adjustment, migration characteristics such as number of parents migrated, age of children at parental migration, duration of parental migration, should be taken into account when we evaluate the impact of migration on children's psychological adjustment.

Whereas, parental migration may create challenges for child development, we may adopt a strength-based approach to understand factors that may be important for left-behind children to sustain adaptive development. In the past decades, researchers have proposed some strength-based frameworks (e.g., the Five Cs model of positive youth development, PYD) to describe, explain, and optimize the development of children who are residing in challenging contexts. According to the Five Cs model of PYD (Lerner, 2015), all youth have strength. The core hypothesis of the Five Cs model of PYD is that, if the strengths of youth (e.g., having hope for the future), can be aligned with the resources in their ecological contexts (e.g., adult support), then young people's positive development may be optimized. Exchanges between individual strengths and ecological assets across the life span may introduce change to all levels of ecology (e.g., family, school, community, and the civic society). Accordingly, it is important to explore some key individual and contextual factors that may benefit psychological adjustment for left-behind children.

As one of the most significant individual factors in child development, future orientation is associated with children's psychological adjustment and health risk behaviors (Zhang et al., 2009). Future orientation is “the degree to which a collectivity encourages and rewards future-oriented behaviors such as planning and delaying gratification” (Ashkanasy et al., 2004). Future orientation is a complex multistage process that is described in terms of three major psychological processes, motivation, planning, and evaluation (Nurmi, 1991). Studies showed that future orientation was negatively associated with unfavorable outcomes, including academic failure, delinquent behavior, substance use, and sexual risk behaviors for children and adolescents (Snyder et al., 2000; Zhang et al., 2009). Future orientation served as a significant protective factor for the development of several disadvantaged and vulnerable young populations, such as children affected by HIV/AIDS (Zhang et al., 2009), adjudicated adolescents (Robbins and Bryan, 2004), and youth with eating disorders (Snyder et al., 2000). However, no research is available regarding the effect of future orientation on the psychological adjustment of left-behind children.

Social support is considered as one of the most important social-contextual factors associated with children's psychological adjustment. It refers to the perception and actuality that leads the subject to believe that he or she is cared for and loved, esteemed, and a member of a network of mutual obligations (Cobb, 1976). Previous research found that, compared to the non-left-behind children, left-behind children reported lower levels of social support, particularly emotional support (Luo et al., 2009). However, social support still played an important role in the psychological adjustment and social adaptation of left-behind children (Hu et al., 2008; Liu, 2009). Prior studies showed that perceived or actual social support from non-family members such as peers, relatives, neighbors, school teachers, and other community members was critical for these children's psychological adjustment when their parents were absent (Hu et al., 2008). However, no study has adopted a strength-based approach to examine the influence of social support coupled with future orientation on psychological adjustment among left-behind children.

Whereas, previous cross-sectional studies indicate that parental migration may negatively affect the psychological adjustment of left-behind children, it is still uncertain if and how protective individual and contextual factors may be associated with children's psychological adjustment from a developmental perspective. As such, a longitudinal study is needed to further explore the associations between parental migration and children's psychological adjustment as well as the effects of protective factors on psychological adjustment among left-behind children. In the current longitudinal study, we examined the following research questions: (1) Does the psychological adjustment of rural children differ by the characteristics and patterns of parental migration? (2) After controlling for key demographics and parental migration characteristics, how are future orientation and social support associated with the immediate and subsequent status of psychological adjustment among left-behind children? Do future orientation and social support play different roles in the psychological adjustment of left-behind children compared to non-left-behind children?

## Method

### Participants

A longitudinal study was conducted in two rural counties of Yulin City, Guangxi province in 2010. In the Wave 1 survey, participants were 1164 rural children. After 6 months, the Wave 2 data were collected from 978 of the Wave 1 participants (84% follow-up rate). Children who had substantial missing data (e.g., over 30% of the items) in either Wave 1 or Wave 2 were excluded, resulting in 897 participants (457 females and 416 males) between 10 and 17 years of age (*M* = 14.09, *SD* = 1.40) who were available in both surveys for the analysis. Among these participants, 403 were left-behind children (227 children with two parents migrating and 176 children with one parent migrating) and 485 were comparison children.

### Survey procedures

Two primary schools in Bobai county and two middle schools in Beiliu county, both of which enrolled a large number of left-behind children, were selected as sampling units. After obtaining permission from the school principals, 16 well-trained interviewers (including one university teacher and 15 psychology graduate students) provided participants with a detailed description of the study design and consent procedure, and then invited them to participate in the study. The eligibility criteria for left-behind children included the following: (1) they were born and raised in the countryside; and (2) one or both of their parents migrated to cities for jobs. Similar numbers of non-left-behind children were recruited from the same schools for comparison. Eligible students provided written informed consent for their participation. We did not obtain written parental consent for participants for the following reasons: (1) Parental consent process was not required by the ethics review board for the study of this nature at the time when we submitted the research protocol; (2) The survey did not include questions that would solicit sensitive personal information regarding the participants, their parents, or relationships within their family; (3) When we explained the consent process to the youth, we mentioned that they can stop taking the survey or skip any questions whenever they do not feel comfortable answering. Participants who provided written consent were asked to complete a self-administered questionnaire. For younger students in lower grades, interviewers provided assistance by explaining survey items using the local dialect. All study procedures including the consent process were approved by the IRB at Beijing Normal University.

### Measures

#### Demographic characteristics

All participants were asked to provide demographics, including gender, age, grade, parental education levels, and family economic level. A composite family socio-economic status (SES) score was then created by indexing parents' (father and mother) education level and family economic status. The composite SES score ranged from 0 to 3, with a higher score indicating better family SES. Children whose parents (father and mother) had not received any education and whose family economic status was very poor compared with their neighbors were coded 0 in their SES, indicating that they had a low family SES. Children whose parents (father and mother) had higher than primary school education and whose family economic status was at least average compared with their neighbors were scored 3 in their SES, meaning that they had good family SES.

#### Patterns of parental migration

Participants were asked to answer two questions about the whereabouts of their father and mother. There were three options for these two questions: “1 = at home (physically living with you); 2 = working in another province; 3 = working in another city in the same province.” Patterns of parental migration were created based on the answers for these two questions: children with one parent migrating (either mother or father was migrating to another city out of their hometown), children with two parents migrating (both parents were migrating to another city out of their hometown), and comparison children (both parents were at home). We did not create subcategories (identifying paternal migrating and maternal migration, separately) for one parent migrating because we did not have sufficient sample of children under the category of maternal migrating.

#### Characteristics of parental migration

Left-behind children answered the following questions regarding their experience with parental migration: “How old were you when your father/mother migrated?” “How long has your father/mother migrated?” (1 = <1 year, 2 = 1–5 years, 3 = more than 5 years); “How frequently does your father/mother contact you?” (1 = never, 2 = usually, 3 = always); “Who is your current caregiver?” (1 = father or mother, 2 = grandparents, 3 = others); “What is the educational level of your current caregiver?”(1 = primary school or less, 2 = middle school, 3 = high school or higher); and perceived caregivers' attitude toward left-behind children (1 = bad, 2 = good). In the present study, current caregiver refers to adults (biological parents or other adults) who were physically taking care of the left-behind children.

#### Future orientation

Three scales were used to measure the children's future orientation, including Children's Future Expectation Scale (Whitaker et al., 2000), Hopefulness about the Future Scale (Whitaker et al., 2000), and Perceived Control over the Future Scale (Whitaker et al., 2000). The 7-item Children's Future Expectation Scale was used to measure children's certainty that some positive outcomes would actually occur in the future with 5-point response options (1 = “not at all” to 5 = “very much”). In addition, children were asked to rate their hopefulness toward the future with the 4-item Hopefulness about the Future Scale. Perceived self-control over the future was assessed by the 7-item Perceived Control over the Future Scale. The Cronbach's alphas for the three subscales at the baseline of the current study were 0.81, 0.77, and 0.74, respectively.

#### Perceived social support

Social support perceived by rural children was measured using a modified version of the multidimensional Scale of Perceived Social Support (Zimet et al., 1988). The scale included 16 items with 5-point response options (ranging from 1 = “strongly disagree” to 5 = “strongly agree”). A composite score was created by averaging responses to these items. Higher composite scores indicated higher levels of perceived social support from various sources (e.g., family, friends, and teachers). The scale had a Cronbach's alpha of 0.90 in the present study.

#### Life and school satisfaction

Life and school satisfaction were measured using two five-point questions (ranging from 1 = “very dissatisfied” to 5 = “very satisfied”). The questions were: “In general, how satisfied are you with your life?” and “How satisfied are you with your schooling”? A higher score indicated a higher level of satisfaction with life or schooling.

#### Happiness

Happiness was measured using Oxford Happiness Questionnaire (OHQ) short scale (Hills and Argyle, 2002). The OHQ short scale included 8 items with 6-point response options (ranging from 1 = “strongly disagree” to 6 = “strongly agree”). The sample questions included: “I can fit in everything I want to,” “I am well-satisfied about everything in my life,” and “I feel that life is very rewarding.” A composite score was created by averaging all the 8 items with higher scores reflecting greater happiness. The Cronbach's alpha was 0.87 for the scale in the current study.

#### Loneliness

The Children's Loneliness scale was used to assess children's perceived loneliness and social dissatisfaction (Asher et al., 1984). The scale consisted of 16 primary items focusing on children's feelings of loneliness (e.g., “It's hard for me to make friends”) and feelings of social adequacy vs. inadequacy (e.g., “I can find friends when needed”) with 5-point response options (ranging from 1 = “strongly disagree” to 5 = “strongly agree”), while the other 8 items served as “filler” ones focusing on children's hobbies or preferred activities. A composite score was created by averaging responses to all 16 loneliness items, with higher scores indicating higher levels of loneliness. The Cronbach's alpha for the scale was 0.79 in the current study.

### Statistical analyses

First, descriptive statistics were calculated for socio-demographic variables. The differences in individual demographic characteristics among the three groups of rural children were explored using Chi-square (for categorical variables) or ANOVA (for continuous variables).

Second, to answer the first research question, group differences (i.e., children with two-parent migration, children with one-parent migration, and comparison children) in terms of satisfaction, loneliness, and happiness in both waves 1 and 2 were examined using ANOVA.

Finally, to answer the second research question, we used linear regression analysis to examine the cross-sectional and lagged effects of future orientation and social support on psychological adjustment after controlling for several key demographics and characteristics of parental migration that have significant associations with psychological adjustment in Wave 1. These analyses involved a two-step process. First, cross-sectional analysis was conducted to investigate the associations of future orientation and social support with the indicators of psychological adjustment in Wave 1 for left-behind children and comparison children, respectively. Second, longitudinal data were analyzed by examining the prediction of future orientation and social support in Wave 1 for psychological adjustment in Wave 2 for these two groups of children, separately. Although, the separate regression analyses for left-behind children and comparison children do not allow us to compare the prediction effects of social support and future orientation between the two groups of children, we would still be able to examine which factors are common protective factors for both groups of children, and which factors may play unique protective roles in the psychological adjustment of a particular group. All statistical analyses were performed using SPSS for Windows 22.0.

## Results

### Sample characteristics

The attrition analysis showed no significant differences between the longitudinal sample (897 children) and attrition sample (186 children) with respect to Wave 1 demographic characteristics, including gender, grade, parental education level, and family economic status. Demographics analysis (Table 1) showed that the left-behind children sample were older than the comparison sample. More comparison children (11.35%) reported higher maternal educational level (i.e., higher than elementary school education) than children with one-parent migration (5.99%) and children with two-parent migration (4.11%). The three groups of children had no significant differences in the composite SES score.

**Table 1:** Demographic characteristics of the sample.

	**Overall**	**Children with two-parent migration**	**Children with one-parent migration**	**Comparison children**	***F/χ*^2^**	***p*-value**
*N*	897 (100%)	227 (25.31%)	176 (19.62%)	485 (54.07%)	185.41	<0.001
**GENDER (%)**						
Male	420 (47.51%)	114 (50.67%)	83 (47.16%)	223 (46.17%)	1.26	0.53
Female	464 (53.70%)	111 (49.33%)	93 (52.84%)	260 (53.83%)		
Age (M ± *SD*)	14.09 ± 1.40	14.59 ± 1.14	14.16 ± 1.46	13.84 ± 1.42	22.61	<0.001
**EDUCATIONAL LEVEL (%)**						
Primary school	294 (33.11%)	27 (11.89%)	53 (30.11%)	214 (44.12%)	78.14	<0.001
Middle school	594 (66.89%)	200 (88.11%)	123 (69.89%)	271 (55.88%)		
**FATHER'S EDUCATIONAL LEVEL (%)**						
Primary school or lower	339 (39.65%)	84 (37.84%)	75 (44.12%)	180 (38.79%)	6.33	0.18
Middle school	420 (49.12%)	118 (53.39%)	80 (47.06%)	222 (47.84%)		
High school or higher	96 (11.23%)	19 (8.60%)	15 (8.82%)	62 (13.36%)		
**MOTHER'S EDUCATIONAL LEVEL (%)**						
Primary school or lower	428 (50.71%)	121 (55.25%)	84 (50.30%)	223 (48.69%)	12.39	<0.05
Middle school	345 (40.88%)	89 (40.64%)	73 (43.71%)	183 (39.96%)		
High school or higher	71 (8.41%)	9 (4.11%)	10 (5.99%)	52 (11.35%)		
**ECONOMIC STATUS (%)**						
Better	16 (1.91%)	3 (1.38%)	2 (1.22%)	11 (2.41%)	4.79	0.57
Average	652 (77.80%)	174 (79.82%)	133 (81.10%)	345 (75.66%)		
Poorer	155 (18.50%)	36 (16.51%)	28 (17.07%)	91 (19.96%)		
Much poorer	15 (1.79%)	5 (2.29%)	1 (0.61%)	9 (1.97%)		
SES (M ± *SD*)	0.94 ± 0.69	0.91 ± 0.60	0.91 ± 0.63	0.97 ± 0.75	0.92	0.40

### Patterns and characteristics of parental migration and psychological adjustment

Table 2 displayed the difference of psychological adjustment measures by parental migratory patterns in both waves. The results showed that children with two parents migrating reported the lowest level of life satisfaction among these three groups of children in both waves; compared with non-left-behind children, left-behind children (one parent or both parents migrating) had lower scores in school satisfaction in both waves. In Wave 1, children with two-parent migrating reported lower scores in happiness compared to children with one-parent migrating, and higher scores in loneliness than those reported by comparison children. In Wave 2, children with two parents migrating reported lower levels of happiness compared to non-left-behind children. No significant group differences were found in terms of loneliness at Wave 2.

**Table 2:** Psychological adjustment disparities among three groups of rural children at wave 1 and 2 survey.

	**Children with two-parent migrating**	**Children with one parent migrating**	**Comparison children**	***F***	***Post-hoc***
**WAVE 1**					
Life satisfaction	3.33 (0.96)	3.54 (1.06)	3.70 (1.05)	10.01^***^	1 < 2 < 3
School satisfaction	3.14 (1.03)	3.07 (1.06)	3.35 (1.17)	5.23^**^	1, 2 < 3
Happiness	3.99 (0.74)	4.15 (0.77)	4.13 (0.80)	3.13^*^	1 < 2
Loneliness	3.46 (0.49)	3.52 (0.48)	3.38 (0.63)	4.62^*^	2 > 3
**WAVE2**					
Life satisfaction	3.35 (0.97)	3.49 (1.05)	3.71 (1.09)	9.14^***^	1 < 2 < 3
School satisfaction	3.04 (1.11)	3.09 (1.04)	3.39 (1.17)	8.36^**^	1, 2 < 3
Happiness	3.96 (0.79)	4.04 (0.74)	4.14 (0.81)	4.09^*^	1 < 3
Loneliness	2.19 (0.62)	2.23 (0.64)	2.13 (0.65)	1.64	

* *p < 0.05*,

** *p < 0.01*,

*** *p < 0.001*.

### Future orientation, social support, and psychological adjustment

Future orientation and social support were included in multilevel regression models to explore their associations with indictors of psychological adjustment for left-behind children and comparison children in both cross-sectional and longitudinal analysis. Prior to the regression analysis, we conducted preliminary analysis to identify key characteristics of parental migration for left-behind children. The preliminary analysis showed that, duration of paternal migration, duration of maternal migration, and education level of current caregiver are key characteristics that may be associated with psychological adjustment. As such, in the regression models for both cross-sectional and longitudinal analysis among left-behind children, we controlled for the key demographics and migration-related characteristics reported in Wave 1. In the regression analysis for comparison children, only key demographics were controlled for.

In the cross-sectional analysis (Table 3), multiple linear regressions for left-behind children showed that, social support (β = 0.20, *p* < 0.001, η^2^ = 0.04) and hopefulness about future (β = 0.13, *p* < 0.05, η^2^ = 0.02) significantly predicted life satisfaction; social support (β = 0.21, *p* < 0.001, η^2^ = 0.05) and control over the future (β = 0.15, *p* < 0.01, η^2^ = 0.02) significantly predicted school satisfaction; future expectation (β = 0.27, *p* < 0.001, η^2^ = 0.13), control over the future (β = 0.12, *p* < 0.05, η^2^ = 0.01) and social support (β = 0.20, *p* < 0.001, η^2^ = 0.04) significantly predicted happiness. Multiple linear regressions for comparison children showed that, future expectation and hopefulness about future significantly predicted life satisfaction (β_*1*_ = 0.15, *p*_*1*_ <0.001, η_*1*_^2^ = 0.04; β_*2*_ = 0.12, *p*_*2*_ < 0.05, η_*2*_^2^ = 0.01) and school satisfaction (β_*1*_ = 0.11, *p*_*1*_ < 0.05, η_*1*_^2^ = 0.01; β_*2*_ = 0.12, *p*_*2*_ < 0.01, η_*2*_^2^ = 0.03); future expectation (β = 0.39, *p* < 0.001, η^*2*^ = 0.20) and social support (β = 0.20, *p* < 0.001, η^*2*^ = 0.04) significantly predicted happiness. Loneliness was not predicted by any of covariate or independent variables among either group of children.

**Table 3:** Results of multilevel regression models for future orientation and social support on psychological adjustment (Cross-sectional analysis).

**Paths**	**Left-behind children**				**Comparison children**			
	***B***	**β**	**Δ*R^2^***	***R^2^***	***B***	**β**	**Δ*R^2^***	***R^2^***
**REGRESSION MODEL FOR W1 LIFE SATISFACTION**								
**Step 1: Demographic characteristics**								
Gender	−0.03	−0.02			−0.02	−0.02		
Age	−0.16	−0.20^***^	0.06^**^	0.06	−0.17	−0.26	0.07^**^	0.07
**Step 2: Migration-related characteristics**								
Duration of paternal migration	−0.05	−0.08			–	–		
Duration of maternal migration	−0.02	−0.03			–	–		
Education level of caregiver	0.05	0.04	0.02	0.07	–	–	–	–
**Step 3: Independent variables**								
Social support	0.24	0.20^***^	0.04^**^	0.11				
Hopefulness about future	0.21	0.13^*^	0.02^*^	0.13	0.20	0.12^*^	0.01^*^	0.08
Future expectation					0.21	0.15^**^	0.04^**^	0.12
**REGRESSION MODEL FOR W1 SCHOOL SATISFACTION**								
**Step 1: Demographic characteristics**								
Gender	0.07	0.03			0.14	0.06		
Age	−0.21	−0.25^***^	0.08^**^	0.08	−0.18	−0.22^***^	0.07^**^	0.07
**Step 2: Migration-related characteristics**								
Duration of paternal migration	−0.12	−0.22^*^			–	–		
Duration of maternal migration	0.03	0.05			–	–		
Education levels of caregiver	−0.07	−0.06	0.03^*^	0.11	–	–	–	–
**Step 3: Independent variables**								
Social support	0.27	0.21^***^	0.05^**^	0.16				
Control over the future	0.25	0.15^**^	0.02^**^	0.18				
Hopefulness about future					0.21	0.12^*^	0.03^**^	0.09
Future expectation					0.18	0.11^*^	0.01^*^	0.10
**REGRESSION MODEL FOR W1 HAPPINESS**								
**Step 1: Demographic characteristics**								
Gender	−0.04	−0.03			0.04	0.02		
Age	−0.10	−0.16^**^	0.02^*^	0.02	−0.07	−0.12^**^	0.03^**^	0.03
**Step 2: Migration-related characteristics**								
Duration of paternal migration	−0.01	−0.03			–	–		
Duration of maternal migration	−0.00	−0.01			–	–		
Education level of caregiver	−0.05	−0.05	0.00	0.02	–	–	–	–
**Step 3: Independent variables**								
Future expectation	0.29	0.27^***^	0.13^**^	0.15				
Social support	0.18	0.20^***^	0.04^**^	0.19	0.20	0.20^***^	0.04^**^	0.07
Control over the future	0.15	0.12^*^	0.01^*^	0.20				
Future expectation					0.42	0.39^***^	0.20^**^	0.26

*In the regression analysis for cross-sectional data of left-behind children, we used W1 life satisfaction, school satisfaction, happiness, and loneliness as dependent variables. W1 future expectation, hopefulness about future, control over the future, and social support served as the independent variables and were taken into stepwise analysis after demographic characteristics (included age, gender and educational level) and migration-related characteristics (duration of paternal/maternal migration, and education level of caregiver) were controlled for. In the regression analysis for cross-sectional data of comparison children, only demographic characteristics were controlled for because migration-related characteristics were not applicable to this group of children. Independent variables that did not have significant effects were not presented in this table*.

* *p < 0.05*,

** *p < 0.01*,

*** *p < 0.001*.

In the longitudinal analyses (Table 4), after controlling for key demographics and characteristics of parental migration at Wave 1 for left-behind children, Wave 1 future expectation positively predicted Wave 2 life satisfaction (β = 0.20, *p* < 0.001, η^2^ = 0.04), school satisfaction (β = 0.16, *p* < 0.05, η^2^ = 0.03), happiness (β = 0.35, *p* < 0.001, η^2^ = 0.12), and negatively predicted loneliness (β = −0.29, *p* < 0.001, η^2^ = 0.09); Wave 1 hopefulness about future was negatively associated with Wave 2 loneliness (β = −0.16, *p* < 0.05, η^2^ = 0.02); Wave 1 social support significantly predicted Wave 2 school satisfaction (β = 0.14, *p* < 0.05, η^2^ = 0.02). In the longitudinal analyses for comparison children, after controlling for key demographic characteristics, Wave 1 future expectation positively predicted Wave 2 life satisfaction (β = 0.15, *p* < 0.01, η^2^ = 0.02), school satisfaction (β = 0.13, *p* < 0.01, η^2^ = 0.02), happiness (β = 0.27, *p* < 0.001, η^2^ = 0.09), and negatively predicted loneliness (β = −0.22, *p* < 0.001, η^2^ = 0.08); Wave 1 hopefulness about future was negatively associated with Wave 2 loneliness (β = −0.15, *p* < 0.01, η^2^ = 0.02); Wave 1 social support significantly predicted Wave 2 happiness (β = 0.14, *p* < 0.01, η^2^ = 0.02).

**Table 4:** Results of multilevel regression models for future orientation and social support on psychological adjustment (Longitudinal analysis).

**Paths**	**Left-behind Children**				**Comparison children**			
	***B***	**β**	**Δ*R^2^***	***R^2^***	***B***	**β**	**Δ*R^2^***	***R^2^***
**REGRESSION MODEL FOR W2 LIFE SATISFACTION**								
**Step 1: Demographic characteristics**								
Gender	−0.07	−0.03			−0.05	−0.02		
Age	−0.11	−0.13^*^	0.03^*^	0.03	−0.17	−0.22^***^	0.06^***^	0.06
**Step 2: Migration-related characteristics**								
Duration of paternal migration	−0.18	−0.33^**^			–	–		
Duration of maternal migration	0.14	0.27^*^			–	–		
Education level of caregiver	0.16	0.14^*^	0.05^*^	0.07	–	–	–	–
**Step 3: Independent variables**								
W1 Future expectation	0.28	0.20^**^	0.04^**^	0.11	0.25	0.15^**^	0.02^*^	0.08
**REGRESSION MODEL FOR W2 SCHOOL SATISFACTION**								
**Step 1: Demographic characteristics**								
Gender	−0.05	−0.02			−0.06	−0.03		
Age	−0.17	−0.18^**^	0.04^**^	0.04	−0.17	−0.20^***^	0.05^***^	0.05
**Step 2: Migration-related characteristics**								
Duration of paternal migration	−0.25	−0.40^**^			–	–		
Duration of maternal migration	0.16	0.26^*^			–	–		
Education levels of caregiver	0.01	0.01	0.06^**^	0.10	–	–	–	–
**Step 3: Independent variables**								
W1 Future expectation	0.25	0.16^*^	0.03^**^	0.13	0.25	0.13^**^	0.02^**^	0.07
W1 Social support	0.21	0.14^*^	0.02^*^	0.15				
**REGRESSION MODEL FOR W2 HAPPINESS**								
**Step 1: Demographic characteristics**								
Gender	−0.10	−0.06			−0.07	−0.04		
Age	−0.07	−0.12	0.01	0.01	−0.13	−0.22^***^	0.07^***^	0.07
**Step 2: Migration-related characteristics**								
Duration of paternal migration	−0.05	−0.13			–	–		
Duration of maternal migration	0.04	0.11			–	–		
Education level of caregiver	0.01	0.01	0.01	0.02	–	–	–	–
**Step 3: Independent variables**								
W1 Future expectation	0.37	0.35^***^	0.12^**^	0.14	0.29	0.27^***^	0.09^***^	0.16
W1 Social support					0.14	0.14^**^	0.02^**^	0.18
**REGRESSION MODEL FOR W2 LONELINESS**								
**Step 1: Demographic characteristics**								
Gender	0.05	0.04			0.01	0.01		
Age	−0.04	−0.07	0.00	0.00	0.05	0.10^*^	0.02^**^	0.02
**Step 2: Migration-related characteristics**								
Duration of paternal migration	−0.02	−0.05			–	–		
Duration of maternal migration	0.04	0.12			–	–		
Education level of caregiver	0.04	0.05	0.01	0.01	–	–	–	–
**Step 3: Independent variables**								
W1 Future expectation	−0.19	−0.29^**^	0.09^**^	0.09	−0.19	−0.22^***^	0.08^***^	0.10
W1 Hopefulness about future	−0.17	−0.16^*^	0.02^*^	0.11	−0.15	−0.15^**^	0.02^**^	0.12

*In the regression analysis for longitudinal data of left-behind children, we used W2 life satisfaction, school satisfaction, happiness, and loneliness as dependent variables. W1 future expectation, hopefulness about future, control over the future, and social support served as the independent variables and were taken into stepwise analysis after demographic characteristics (included age, gender and educational level) and migration-related characteristics (duration of paternal/maternal migration, and education level of caregiver) were controlled for. In the regression analysis for longitudinal data of comparison children, only demographic characteristics were controlled for because migration-related characteristics were not applicable to this group of children. Independent variables that did not have significant effects were not presented in this table*.

* *p < 0.05*,

** *p < 0.01*,

*** *p < 0.001*.

## Discussion

The current study presented both cross-sectional and longitudinal evidence to suggest that, left-behind children are disadvantaged in terms of unfavorable status of psychological adjustment compared with children living with both of their parents in the same rural communities in Guangxi province. In addition, we found that future orientation and social support can serve as protective factors for some indicators of immediate and subsequent status of psychological adjustment among left-behind children and comparison children. Future orientation plays similar roles in the psychological adjustment of both left-behind children and comparison children. Compared to non-left-behind children in rural areas, social support appears to be more important for the immediate status of psychological development of left-behind children.

Our analyses revealed that left-behind children reported lower levels of life satisfaction, school satisfaction and happiness, as well as a higher level of loneliness in both waves, compared to rural children living with both of their parents. The finding is consistent with previous studies, which indicated that parental migration had a significantly negative effect on psychological adjustment of left-behind children, especially children with two migrating parents (Fan and Sang, 2005; Ma et al., 2008). Therefore, the unfavorable status of psychological adjustment of left-behind children requests attention and intervention efforts from researchers and practitioners.

Another important feature of the present study is the testing of the protective effects of future orientation and social support on the immediate and subsequent status of psychological adjustment among left-behind children and comparison children. Both cross-sectional and lagged effects analyses showed that one or more sub-measures of future orientation served as important protective factors for indicators of psychological adjustment of all rural children, including left-behind children and non-left-behind children. Specifically, for both groups of children, higher scores in hopefulness about future was related to higher levels of life satisfaction, and higher scores of future expectation was associated with higher levels of happiness measured at Wave 1. Left-behind children who reported higher scores in control over the future were likely to exhibit higher levels of school satisfaction and happiness measured at the same time. Comparison children who reported higher scores of future expectation were likely to have higher levels of school satisfaction measured at the same time. For both groups of children, future expectation is beneficial to all indicators of psychological adjustment measured subsequently among both groups of children; hopefulness about future is also an important protective factor for loneliness measured subsequently. These findings further highlight the protective effects of future orientation on children's psychological adjustment and elevate our understanding of the specific effects of each indicator of future orientation. It should be noted that, rural children's higher levels of hopefulness about future and future expectation may relate to lower vulnerability to loneliness, a typical emotional problem among rural children, especially left-behind children (Su et al., 2013; Ai and Hu, 2016). Interventions for the promotion of psychological adjustment among left-behind children should foster future orientation, especially future expectation, to improve their current and subsequent psychological adjustment.

In addition, we found that social support is also an important protective factor for the psychological adjustment of rural children, especially for left-behind children. For comparison children, higher scores in social support were related to higher levels of happiness measured at the same time, and a better status of school satisfaction measured subsequently. Social support was associated with life satisfaction, school satisfaction, and happiness among left-behind children at the cross-sectional level, as well as school satisfaction reported 6 months later. Cumulative evidence from a variety of settings have showed that a social support network can help disadvantaged, troubled, or vulnerable youth to cope with difficulties and, as well, facilitate their psychological development (Cohen and Wills, 1985; Dalgard et al., 1995; Zhao et al., 2008; Lakey and Orehek, 2011). The current study significantly contribute to the research field by examining the cross-sectional and longitudinal effects of social support on psychological adjustment among left-behind children, respectively. Given that social support is related to one or more indicators of psychological adjustment measured at the same time or subsequently, and more associations exist between social support and psychological adjustment at the cross-sectional level compared to that in the longitudinal analysis, a highly responsive social support network may be essential to the psychological adjustment of rural children, especially left-behind children.

Several potential limitations of the present study are worth noting. First, despite efforts to ensure sample representation, the participants were only chosen from two rural counties in China. Thus, the findings may not be generalized to left-behind children in other areas of the country. Second, although the current two-wave longitudinal study has deepened our understanding of the associations between positive factors and psychological adjustment among left-behind children, future longitudinal studies using data from multiple time points are still needed to further explore how may the coactions of protective individual and contextual factors mitigate the adverse effect of parental migration and shape the development of left-behind children. Third, the present study solely relied on self-assessment of key psychological adjustment variables. A better measurement scheme should be utilized to gather information from left-behind children, as well as from other sources, including reports from parents, current caregivers, teachers, and peers. Finally, although the separate regression analyses allowed us to preliminary compare the roles of future orientation and social support in the psychological adjustment of left-behind children and comparison children respectively, we were not able to compare the effects of these protective factors between these two groups of children because they were not sharing the same sets of covariates in the present study. Future research may adopt appropriate approach to compare the effects of these protective factors between the two groups of children.

Despite these potential limitations, the findings of the present study have some important implications for psychological health promotion and future research. First, the findings highlight the need for governments and local communities to recognize the adverse effect of parental migration on the psychological adjustment of left-behind children. As such, effective psychological adjustment promotion and intervention efforts should be provided to vulnerable rural children, especially for children with two migrating parents. Second, the government and concerned community organizations may explore effective ways to help rural children build a positive perspective toward their future and enhance the social support network for them. Governments and local communities may seek effective intervention strategies from some successful programs designed for other vulnerable Chinese children. For example, in the resilience-based intervention research for children affected by parental HIV (Li et al., 2017), researchers designed activities to facilitate positive development of these children. These activities include discussions about building positive future orientation, seeking efficient social support, and emotion regulation and so on (Li et al., 2017). In sum, researchers and practitioners should understand what and how protective factors influence psychological adjustment, and develop effective intervention programs to enhance psychological adjustment of left-behind children.

## Author contributions

DL is the primary investigator of the study and provided comments and ideas, and revised this paper. SS did data analysis, developed and revised the manuscript. XL provided comments and ideas, and helped revised the manuscript. MZ helped conduct the study, including developing survey, sampling, data analysis, and proof reading the manuscript.

### Conflict of interest statement

The authors declare that the research was conducted in the absence of any commercial or financial relationships that could be construed as a potential conflict of interest.
